# 383. Pharmacokinetics and Safety in Healthy Adults of RSM01, a Novel RSV Monoclonal Antibody, and Population PK Modeling to Support Pediatric Development

**DOI:** 10.1093/ofid/ofad500.453

**Published:** 2023-11-27

**Authors:** Micha Levi, Shayne Watson, Aparna B Anderson, Dale Taylor, Joleen T White, Luisa M Stamm, Michael W Dunne

**Affiliations:** Bill & Melinda Gates Medical Research Institute, Cambridge, Massachusetts; Bill & Melinda Gates Medical Research Institute, Cambridge, Massachusetts; Bill and Melinda Gates Medical Research Institute, Glastonbury, Connecticut; PPD, part of Thermo Fisher Scientific, Orlando, Florida; Bill & Melinda Gates Medical Research Institute, Cambridge, Massachusetts; Bill & Melinda Gates Medical Research Institute, Cambridge, Massachusetts; Bill & Melinda Gates Medical Research Institute, Cambridge, Massachusetts

## Abstract

**Background:**

RSV is a common cause of infant respiratory infection and hospitalization. RSM01 is a novel anti-RSV mAb targeting the F glycoprotein essential for viral entry with a YTE mutation for half-life extension. Phase 1a PK and safety results are presented, along with an adult population PK model used to predict pediatric exposures via simulation.

**Methods:**

In this double-blind Phase 1a study, healthy males and non-childbearing females aged 18-49 yrs were randomized 6:1 within dose cohorts to receive a single dose of RSM01 or placebo (PBO) in the following RSM01 dose sequence: 300mg IV, 300mg IM or 1000mg IV, 3000mg IV and cohort at 600mg IM. Serial serum samples were taken to measure RSM01 PK using a validated immunoassay method. A noncompartmental analysis (NCA) was conducted, and a population PK model was developed to characterize adult PK and predict pediatric PK using allometric scaling. Pediatric RSM01 PK was predicted for African infants at different IM doses.

**Results:**

Overall, 56 participants received RSM01 (n=48) or PBO (n=8): median age 30 yrs (range 19-48), 52% male (n = 29), 66% White (n=37), median BMI 24.6 kg/m^2^ (range 18.1-29.4). 12/48 (25%) RSM01 recipients and 2/8 (25%) PBO recipients reported an AE, with no hypersensitivity reactions, serious AEs, or deaths reported. The median T_max_ was ∼6–7 days after IM injection. C_max_ and AUC_last_ increased dose proportionally following IV administration. RSM01 adult PK was characterized using a two-compartment model with first-order elimination. IM absorption was described using a zero-order rate constant. Inter-individual variability was included for clearance (CL), central and peripheral volumes (V2 and V3). The RSM01 model estimated that distribution and a terminal half-life were 1.3 and 79.1 days, respectively, and IM bioavailability was ∼82%. The model predicted that following a 50 mg IM dose, median serum RSM01 concentration on day 150 in African infants 0-12 months old to be ∼ 28 mcg/mL, higher than the target exposure based on preclinical models (6.8 mcg/mL).

Serum RSM01 NCA PK summary

**Figure:**
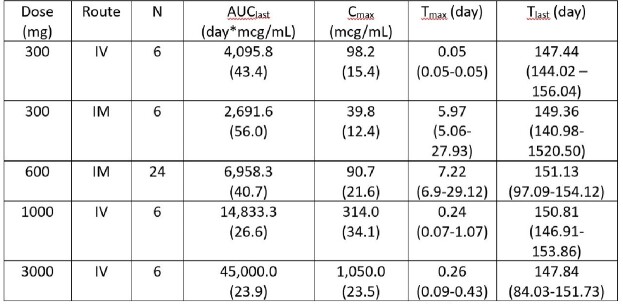

Note: AUClast and Cmax values are presented as geometric mean (CV). Tmax and Tlast are presented as median (min-max).

Arithmetic Mean (±SD) Serum RSM01 Concentration-Time Curve by Treatment

**Figure:**
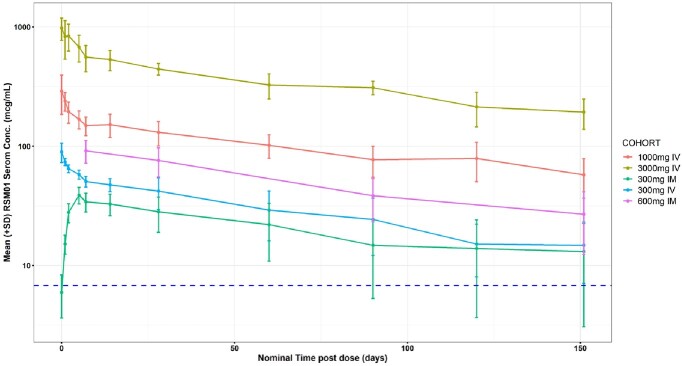

Note: Horizontal blue dashed line represents the target exposure based on preclinical models (6.8 mcg/mL).

**Conclusion:**

RSM01 was shown to be well tolerated in healthy adults. Its long half-life and the predicted infant PK profiles support further pediatric development and indicate the potential for dosing once every RSV season.

**Disclosures:**

**Micha Levi, PhD**, Bill & Melinda Gates Medical Research Institute: Salary as a full-time employee **Shayne Watson, MSc**, Bill & Melinda Gates Medical Research Institute: Salary as a full-time employee **Dale Taylor, MD**, Sanara Medtech Inc: Stocks/Bonds **Luisa M. Stamm, MD, PhD**, Bill & Melinda Gates Medical Research Institute: Salary as a full-time employee **Michael W. Dunne, MD**, Bill & Melinda Gates Medical Research Institute: Salary as a full-time employee

